# CLIP-Driven Prototype Network for Few-Shot Semantic Segmentation

**DOI:** 10.3390/e25091353

**Published:** 2023-09-18

**Authors:** Shi-Cheng Guo, Shang-Kun Liu, Jing-Yu Wang, Wei-Min Zheng, Cheng-Yu Jiang

**Affiliations:** College of Computer Science and Engineering, Shandong University of Science and Technology, Qingdao 266590, China; guoshicheng@sdust.edu.cn (S.-C.G.); liushangkun97@163.com (S.-K.L.); jingyuw994@gmail.com (J.-Y.W.); jcyu6356789@gmail.com (C.-Y.J.)

**Keywords:** few-shot semantic segmentation, few-shot learning, semantic segmentation, multi-modal, CLIP

## Abstract

Recent research has shown that visual–text pretrained models perform well in traditional vision tasks. CLIP, as the most influential work, has garnered significant attention from researchers. Thanks to its excellent visual representation capabilities, many recent studies have used CLIP for pixel-level tasks. We explore the potential abilities of CLIP in the field of few-shot segmentation. The current mainstream approach is to utilize support and query features to generate class prototypes and then use the prototype features to match image features. We propose a new method that utilizes CLIP to extract text features for a specific class. These text features are then used as training samples to participate in the model’s training process. The addition of text features enables model to extract features that contain richer semantic information, thus making it easier to capture potential class information. To better match the query image features, we also propose a new prototype generation method that incorporates multi-modal fusion features of text and images in the prototype generation process. Adaptive query prototypes were generated by combining foreground and background information from the images with the multi-modal support prototype, thereby allowing for a better matching of image features and improved segmentation accuracy. We provide a new perspective to the task of few-shot segmentation in multi-modal scenarios. Experiments demonstrate that our proposed method achieves excellent results on two common datasets, PASCAL-$5^{i}$ and COCO-$20^{i}$.

## 1. Introduction

In recent years, there have been significant advancements in semantic segmentation on various large-scale datasets [1,2] due to the continuous development of various deep learning networks [3,4]. However, this task requires a large number of pixel-level labels to train the model, which makes it costly and time consuming. Moreover, the trained model has almost no recognition ability for classes that have not been seen during training.

The purpose of few-shot semantic segmentation (FSS) is to segment a new class using only a few support samples, while the query images are unseen before. The challenge of this task is to train a model that can learn features of the available classes and also generalize to the unavailable classes with only a few samples in the training set. In FSS methods [5,6,7], the main approach is to use prototypes to represent each class and then match the prototypes and query images by elaborate matching methods. “Prototype” is an abstract concept; briefly, it represents the average representation of a class, which can be obtained by aggregating image features through clustering methods. To utilize a prototype for guiding the segmentation of query images, researchers have proposed several methods to make prototypes more representative for the target class. For instance, in [8], a prototype alignment method is proposed for FSS tasks with the goal of enhancing the expressive ability of a single prototype. Ref. [9] extends a single-class prototype to multiple to cope with the variable appearance of class and different scenarios. Although the above methods have made great progress in FSS, only image features are employed in the prototype generation process, and no additional modal features are considered. Ref. [10] shows that cross-modal training is a lightweight and effective approach for adapting pretrained multi-modal models to downstream unimodal tasks. We believe that cross-modal features can be useful in few-shot segmentation as well, and that incorporating text features in the prototype generation process can make the class prototypes more representative.

A increasing number of studies have shown that multi-modal models [11,12,13] based on text-image features perform well in image classification and image retrieval tasks. The milestone work CLIP [14] proved that training methods using text–image pairs can yield models with excellent migration and generalization capabilities on traditional vision tasks. Using CLIP for different downstream tasks requires only simple fine-tuning to achieve high performance [15,16,17]. Yet compared to image classification, the challenge of semantic segmentation is to correctly classify each pixel. CLIP learns high-level semantic correlations between images and text rather than pixel-level information. Undoubtedly, simply fine-tuning CLIP to adapt to semantic segmentation tasks is not feasible. Therefore, researchers have attempted various methods to enable CLIP to perform these tasks. The “encoder–decoder” paradigm is recognized as one of the most useful ways for semantic segmentation. This paradigm is also mostly used for clip-based dense prediction tasks [18,19,20]. Well-designed loss functions and fine-tuning methods enable CLIP to be adapted to such tasks. Existing approaches [21,22] use text features as weights for classifiers or design prompt engineering such as learnable tokens to replace manual templates. Nonetheless, the parameters in CLIP is enormous, and fine tuning it with few support images can easily cause over-fitting. Therefore, we combine CLIP with an FSS model based on the prototype structure. The generation of prototypes is an essential procedure in the FSS task, since each pixel is classified by computing the cosine similarity or Euclidean distance between the prototype and sample features. Our work investigates the fusion of text features with image features and incorporates them into the process of prototype generation. Building on previous work, we involve both text and images as training samples in the training of the model instead of treating the text features as weights of the classifier without participating in the training process.

**Figure 1:**
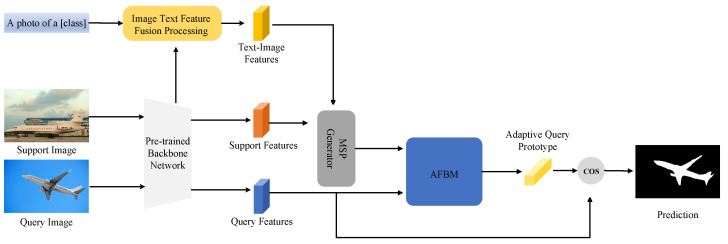
Overview of our proposed network for 1-shot segmentation. We optimize the process of generating prototypes using fusion features of image and text. Our framework consists of MSP and an AFBM module. Given only one annotated training image, our network is able to segment test images with new classes.

In this paper, we propose a multi-modal few-shot segmentation method based on the prototype structure. Referring to Figure 1, our approach consists of three main parts. The first part is to involve the text samples in the model training instead of freezing the text encoder of CLIP. We use a very simple method to involve text samples in the training process and without extra training time. The second part is Multi-modal Support Prototype (MSP) Generator; this module integrates text features and image features and adds the fused features to the prototype generation process. MSP maps two modalities to the same high-dimensional semantic space, making the prototype more representative of the object class. In the last part, to make the prototype better match image features, we design a new matching strategy called the Adaptive Foreground Background Matching (AFBM) module. The AFBM module utilizes MSP to combine the foreground and background information of the query image features. This module generates adaptive query prototypes using query image features, although previous work such as PANet [8] and CRNet [23] has also explored query feature prototypes they do not involve text features and image foreground and background information. To match the proposed adaptive query prototype and multi-modal support prototype, we designed new loss functions to further exploit various prototypes. In general, our contributions include the following:

- Our work combines CLIP with a few-shot semantic segmentation model based on a prototype structure. This approach addresses the problem of over-fitting when fine-tuning CLIP uses a few support images.
- We propose MSP that involves text samples in model training and introduce image text fusion features in the prototype generation process. Multi-modal support prototypes are better at representing the same semantic information of an image and text compared to single-modal prototype features for representing an object class.
- We propose the AFBM module, which uses the foreground and background information of an image combined with query image features and MSP to generate an adaptive query prototype. Experiments demonstrate the excellent performance of our method on diverse datasets.

## 2. Related Work

### 2.1. Semantic Segmentation

Semantic segmentation is an important task in computer vision, which aims to assign each pixel in the input image to the corresponding class. The proposed Full Convolutional Network (FCN) [24] marks the beginning of researchers’ application of CNN to pixel-level prediction. Unlike previous traditional works [25,26], FCN replaces the fully connected layer with a convolutional layer and upsamples the feature map using deconvolution. The feature map is restored to the same size as the input image so that predictions can be made for each pixel. Researchers have proposed various methods to improve the accuracy of prediction and to make full use of pixel information. In recent years, the main approaches for semantic segmentation have been based on encoder–decoder structures [27,28,29], which use pyramid structures to extract features at multiple scales or attention mechanisms to weight important information. These approaches aim to increase the perceptual field while maintaining feature resolution. The U-Net [27] follows the “encoder–decoder” architecture, which involves the use of multiple convolutional and pooling layers in the encoder to extract image features and aggregate high-dimensional information. The decoder, on the other hand, uses a combination of upsampling and convolutional layers to restore the feature map to its original size and generate pixel-level results. After U-Net, the DeepLab series [30,31,32] which uses dilated convolution and multi-scale feature fusion to further improve the segmentation accuracy. Attention U-Net [33] introduces an attention mechanism that suppresses irrelevant regions in the input image while highlighting salient features in specific local regions.

Recently, transformer has developed rapidly in the field of computer vision. Since the emergence of VIT [34], many subsequent works have used transformer as the backbone of the model. SETR [35] proved the feasibility of transformer in image segmentation, which is followed by a series of work such as Segmenter [36] and SegFormer [37]. These methods explored more possibilities of transformer in the field of semantic segmentation. Despite the great success of the above approach, there are still some problems: the long training time of a model implies that more computational resources are required as the number of parameters increases. The model does not generalize well to unseen classes, and it also requires a large number of accurate pixel-level annotations to train effectively.

### 2.2. Few-Shot Learning

Few-shot learning aims to enable the model to learn and generalize with a few labeled data, allowing the model to recognize previously unseen classes. The existing methods can be divided into three groups: transfer-learning method, data augmentation-based method, and metric-based method. The transfer learning-based methods [38,39,40] typically involve pre-training a model on a large datasets and then fine-tuning some of its layers on smaller, targeted datasets. Weiyu Chen et al. [38] proposed a two-stage training method, first using the basic class to train the model and then fine-tuning the model to improve the generalization ability of the model on the novel class. However, this process can be time-consuming due to the need for both pretraining and fine-tuning phases. The second approaches are the data augmentation-based methods [41,42,43]. Since there are few labels available for small-sample learning, researchers aim to increase the diversity of samples with limited labels through data augmentation, which can expand the number of each category. Ref. [44] proposed an automatic encoder, using the encoder to obtain the deformation information between two samples of the same category and using it to expand the samples of the new category so as to achieve the effect of data enhancement. However, this approach cannot fully address the issue of class imbalance. The metric-based methods [45,46,47] are inspired by meta-learning, which provide a paradigm for gaining experience through multiple learning stages and using that experience to improve its next learning performance. In our work, we use support–query pairs to generate prototype features to measure correlations.

### 2.3. Few-Shot Semantic Segmentation

Few-shot semantic segmentation (FSS) is a challenging task in computer vision. The goal is to enable models to perform segmentation with a small number of training samples. Additionally, the models should be able to recognize novel classes not present in the training set. This means that the model requires strong migration and generalization capabilities. To address the problem of generalization to the novel class, researchers have proposed various methods based on few-shot learning. Amirreza Shaban et al. [48] first proposed the FSS task in OSLSM [48] and designed a two-branch network where the first branch is used to receive the labeled images from the support set, and the other branch is used to receive the images to be segmented in the query set. The two-branch network structure became the main paradigm for subsequent studies on this task.

In order to identify the images in the query set, there are two main approaches. One approach aggregates image features to generate prototypes and then uses metric functions such as cosine similarity and Euclidean distance for metrics and classification. The PLNet [49], proposed by Nanqing Dong et al. [49], is the first FSS framework to introduce prototype learning. It measures the similarity between prototypes and query features using a metric function. Kaixin Wang et al. [8] proposed a prototype alignment method that enhances the feature aggregation capability of individual prototypes during training. In this way, the generated prototype is more similar to the features of the query images. SG-One [50] was proposed by Xiaolin Zhang el al. [50]. They use masked average pooling (MAP) to extract the representation vectors of the targets in the support set. MAP is an effective way that combines the features extracted by the backbone network with its ground truth mask to generate prototype features. Its simplicity and effectiveness have led many subsequent works to adopt this approach for prototype generation. According to Qi Fan et al. [51], the pixel similarity between different objects belonging to the same class exhibits a significant gap compared to the pixel similarity between the same objects. To address this issue, they propose to leverage query prototypes to match query features.

Another alternative method is to employ an encoder–decoder network structure. The process involves first using the encoder to encode the features of both the support set and the query set images. Then, an elaborate feature comparison module is used to activate the same class of features in the query images. Finally, the feature decoder is utilized to optimize the comparison results of the previous stage and generate the prediction map. CANet, proposed by Chi Zhang et al. [52], introduces a dense comparison module (DCM) and an iterative optimization module (IOM) to leverage convolution for performing the metric. This approach significantly enhances the segmentation performance compared to previous methodologies. Zhuotao Tian et al. [53] made certain adjustments to the output layer of the backbone network by incorporating the idea of CANet. Specifically, they employed the high-dimensional features originally outputted from the last layer to generate a rough segmentation result, which was then used to guide the network’s training process. Additionally, they introduced a feature enhancement module that leverages features from supporting ensemble images to enhance the query image features. Much of the subsequent work has focused on designing different modules to aggregate features of the two types of images using the support set features and query image features extracted through the backbone network. For example, SD-AANet [54] designs two modules to aggregate fusion features SDPM and SAAM. HSNet [55] aggregates multi-scale features using 4D convolutional kernels.

### 2.4. CLIP in Segmentation

CLIP is a state-of-the-art model developed by OpenAI. It is designed to learn joint representations of images and their associated textual descriptions. By leveraging a large-scale dataset of image–text pairs, CLIP learns to understand the semantic relationships between visual and textual information. Unlike traditional computer vision models that focus solely on images, CLIP takes a multi-modal approach by considering both images and text together. It utilizes a transformer-based architecture, which allows it to capture complex relationships and contextual information across modalities. CLIP is pretrained on a vast amount of internet data, which enables it to acquire a broad understanding of various concepts and objects. During training, CLIP learns to associate images and their corresponding texts, effectively mapping them into a shared embedding space. Yongming Rao et al. [19] demonstrated that CLIP can yield good results in semantic segmentation tasks by modifying the output of its last layer and designing a text–pixel loss function. This enables CLIP to perform dense prediction tasks. Boyi Li et al. [18] extended the use of CLIP to zero-shot image segmentation tasks by incorporating text features with image features in dense prediction transformers (DPT) during the training process. This allowed them to obtain fused features which were then fed into the decoder for image segmentation.

Due to the efficacy of CLIP’s pretraining parameters, many image segmentation approaches use CLIP to generate coarse masks in the first step. These masks are then iteratively trained using carefully designed modules, eventually producing accurate segmentation results. For instance, Haohan Wang et al. [56] proposed the Iterative Mutual Refinement (IMR) module, which is combined with CLIP to generate coarse image masks that are further refined through iterative training. Additionally, Chong Zhou et al. [57] combined the predicted image mask generated by CLIP with other segmentation networks and further trained the model to achieve improved performance. Because of the impressive effectiveness of integrating visual and textual features in the embedding space, numerous methods have emerged that leverage CLIP for performing few-shot segmentation tasks. Timo Lüddecke et al. [58] introduce a lightweight transformer-based decoder that facilitates the interaction between support features, query features, and text features. Mengya Han et al. [59] use CLIP to solve the problem of few-shot part segmentation. They utilize the text encoder to generate text features for each part, which aids in a more efficient learning of visual features. Shuai Chen et al. [60] extracts image and text features by using CLIP and generates a class-agnostic coarse mask. By adopting this class-agnostic process, the network can better balance the influence of different classes during training, leading to more equitable and effective performance.

Although various methods have been employed to modify CLIP for pixel-intensive prediction tasks, the training process still necessitates a relatively large number of training samples and time to attain optimal performance. Our proposed multi-modal FSS method is based on the prototype structure and has achieved good results using only a small number of training samples and a short training time.

## 3. Method

### 3.1. Task Description

The few-shot segmentation task aims to identify previously unseen classes using a limited number of samples, so the dataset is structured differently from previous tasks. Suppose a dataset is divided into a training set $E_{train}$ and a test set $E_{test}$. In traditional segmentation tasks, the training and test sets have the same number of classes. However, in few-shot segmentation tasks, $E_{train}$ and $E_{test}$ do not intersect $\left(E_{train}\cap E_{test}=⌀\right)$. Intuitively, once the model is trained, we select an image from the $E_{test}$ as the query image, which does not belong to any class in the $E_{train}$. Then, we use one or more images from the test set as the support image to segment this new class. We utilize the episodic paradigm approach to train the model, which is a meta-learning-based approach proposed in [61]. This approach was first employed in [48] for few-shot segmentation tasks. The main training process with reference to the previous work can be summarized as follows: *K*-shot support images are selected from the set S of support images {$SI_{1}$,..... .$SI_{k}$} and its corresponding ground truth mask {$MS_{1}$,.... .$MS_{k}$}. A query image $I_{q}$ and its corresponding mask $M_{q}$ are selected from the query image sets Q. The model obtains few-shot segmentation tasks {$S_{i}$, $Q_{i}$} from the training set $E_{train}$ and uses the information in the support set S to guide the segmentation tasks on the query set Q. In the training phase, each few-shot segmentation task is randomly sampled from $E_{train}$ and treated as a distinct task. As a result, the model can be generalized to new few-shot segmentation tasks after training. During the testing phase, the model’s performance is evaluated using the $E_{test}$ dataset, which is consistent with the training phase. The model utilizes information from the support set S to segment the query set images in a guided manner. In the testing phase, $M_{q}$ in the query set Q is not visible to the model.

**Figure 2:**
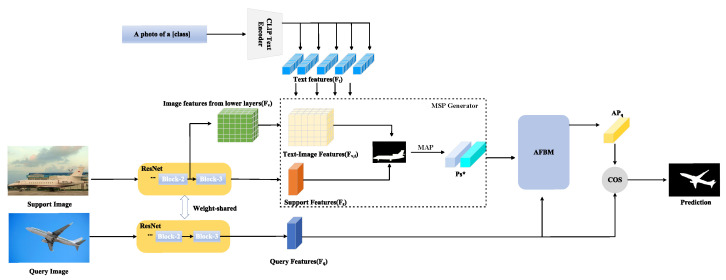
Overview of the network architecture. We first combine the image features with the text features extracted via CLIP to generate a multi-modal support prototype. Then, we utilize AFBM to generate adaptive query prototypes to match query features.

We will present our core ideas (referring to Figure 2) in the following sections. We first combine the image features and text features extracted using CLIP to generate multi-modal support prototypes. This prototype captures the combined information from both images and text, enabling a comprehensive representation of the underlying data. Then, we employ an AFBM module to generate adaptive query prototypes. AFBM utilizes the query features to adaptively generate prototypes that are specifically tailored to match the query images. This adaptive approach enhances the model’s ability to capture relevant information and improve the matching performance.

### 3.2. Image-Text Feature Fusion Processing

In this section, our main idea is to introduce how textual features can be integrated into the network and incorporated into the training process. Adding text features as auxiliary modalities can enhance the model’s capacity to identify new classes. Similar to previous CLIP-based works, a manual prompt template {a photo of a class} is used for various categories where the class in the text needs to be replaced with an image. In contrast to previous approaches, our goal is to enhance the model with novel text features by training on text samples rather than solely relying on text features as weights for the classifier. The current few-shot segmentation model which is based on the prototype structure takes a pair of support images and query images {$I_{s}$, $I_{q}$} as input. These are fed to a backbone network with shared parameters which generates corresponding features {$F_{s}$, $F_{q}$}. Previous studies such as CANet [52] have shown that the deeper layers of a ResNet-based backbone network have a significant impact on final performance. Additionally, PFENet [53] proposed that features from different layers can be utilized to improve model training.

In our approach, we extract mid-level image features $F_{v}$ using the backbone network. Unlike previous few-shot segmentation works, we innovatively combine image features with text features. We believe that incorporating supplementary modalities into the training process can improve the model’s capacity to differentiate among unfamiliar classes resembling how humans obtain new knowledge. In daily life, using images as aids for learning novel knowledge is more effective than relying solely on text. CLIP as a large image text pretraining model has an image encoder $E_{v}$ and a text side encoder $E_{t}$. We input the manual prompt template a photo of a class into the CLIP text encoder $E_{t}$ to obtain the text feature $F_{t}$. We reshape $F_{t}$ into a feature vector of the same size as $F_{v}$ and then combine $F_{v}$ and $F_{t}$ in the feature dimension to create a new image–text fusion feature. To make the feature fusion more adequate, we pass the fused features through a 1 × 1 convolution layer and ***relu*** activation function.

(1) $$\begin{array}{c} F_{v,t}=\mathrm{relu}\left(\mathrm{conv}\left(\mathrm{cat}\left(F_{v},F_{t}\right)\right)\right) \end{array}$$

According to Equation (1), ***cat*** denotes that concatenating $F_{v}$ and $F_{t}$ in the feature dimension. We use ***conv*** to refer to 1 × 1 convolution layer and ***relu*** to denote the activation function. At last, we use the fused features to generate prototypes and perform foreground–background matching, which will be described in the following sections. In Section 4, our experiments demonstrate that the fused features are more effective in generalizing to new categories than using single image features alone.

### 3.3. Multi-Modal Support Prototype Generator

The primary focus of this section is to describe our proposed prototype generation process based on multi-modal fusion features. To begin, we will first outline the process of generating prototype features in most previous works. The image features {$F_{s}$, $F_{q}$} are obtained after feeding the support image and the query image into the parameter-sharing backbone network, and the support prototype can be expressed by Equation (2).

(2) $$\begin{array}{c} P_{s}=\mathrm{MAP}\left(F_{s},M_{s}\right) \end{array}$$

The formula ***MAP*** stands for masked average pooling, and $M_{s}$ represents the ground truth mask of the support image. The generated support prototype $P_{s}$ is used to measure the features of the query image by the cosine similarity function, and then, the predicted mask is obtained by softmax function.

(3) $$\begin{array}{c} {\hat{M}}_{1}=\mathrm{softmax}\left(\mathrm{cosine}\left(P_{s},F_{q}\right)\right) \end{array}$$

According to Equation (3), we use ***cosine*** to refer to the cosine similarity function and ***softmax*** to denote the activation function. In conclusion, the support prototype plays a crucial role in determining the final segmentation result. If it can cover a wider range of accurate semantic information, then the segmentation performance will improve accordingly. Therefore, we aim to incorporate textual features into the support prototype generation process to further enhance the segmentation accuracy. By using both image and text modalities, we can leverage the complementary information between them to generate prototypes that encapsulate a more comprehensive and accurate representation of the underlying semantic information. As a result, this method can enhance the model’s capability to differentiate among distinct object classes and generalize to new classes with only a limited number of labeled examples.

(4) $$\begin{array}{c} P_{s}^{*}=\mathrm{MAP}\left(F_{v,t},M_{s}\right)⊕P_{s} \end{array}$$

We fuse the image text fusion features $F_{v,t}$ obtained in Section 3.2 with the ground truth mask $M_{s}$ of the support images to generate new support prototypes (as in Equation (4)). Previous works generate prototypes by using mask average pooling combined with a ground truth mask and deep features acquired through the backbone. In contrast, we generate prototypes by combining the fused shallow features of the image and text features with the ground truth mask of the support image, which we call MSP. We still utilize the deep features of the image, as they contain crucial high-level semantic information. Giving up these features would adversely affect the final segmentation results. To generate the final support prototype $P_{S}^{*}$, we connect the prototype generated by the support image feature $F_{s}$ with the multi-modal support prototype. In this way, the prototype features cover richer semantic information, and thus, the perception of the novel class is more accurate. The prototype features produced by this method will be used in the foreground–background matching process outlined in Section 3.4.

### 3.4. Adaptive Foreground Background Matching Module

**Figure 3:**
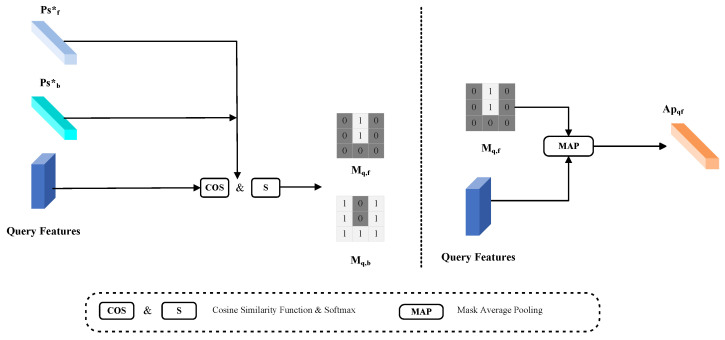
The generation process of adaptive foreground prototype. We obtain the predicted mask by calculating the similarity between the query features and the prototype. Then, we leverage the predicted query mask to aggregate query features to generate an adaptive foreground prototype.

The method for incorporating textual features and the process for generating MSP were introduced in Section 3.2 and Section 3.3. This section focuses on generating foreground and background prototypes using the AFBM module based on the foreground and background information of the image (as shown in Figure 3). These prototypes are then combined with the multi-modal support prototypes to generate the final adaptive query prototype for classifying the query image. Previous work mostly generated support prototype features to segment the query image. However, we argue that generating the query prototype using the query image features can result in better segmentation. We combine the foreground–background prototype and multi-modal support prototype with the query image features to generate the required adaptive query prototype. This prototype is then used to segment the query image and obtain the final segmentation result. Normally, the object we aim to segment is the foreground of an image, whereas the background of the image is often cluttered. Nevertheless, the background pixels also have an impact on the final performance. To improve the final segmentation performance, we propose generating a background prototype by aggregating the background pixels.

(5) $$\begin{array}{c} P_{sf}=\mathrm{MAP}\left(F_{s},M_{s}\left(m==1\right)\right) \end{array}$$

(6) $$\begin{array}{c} P_{sb}=\mathrm{MAP}\left(F_{s},M_{s}\left(m==0\right)\right) \end{array}$$

To assign each pixel in the image’s ground truth mask to a certain class, we specify the label 1 as the foreground pixel and the label 0 as the background pixel. In accordance with Equation (5), we generate the foreground prototype by combining the pixels considered as foreground with the image features through the ***MAP*** function. The background prototype is implemented in the same manner as the foreground. In combination with the multi-modal support prototype discussed in Section 3.3, the final process for generating foreground–background prototypes can be represented by the following equations, Equations (7) and (8).

(7) $$\begin{array}{c} P_{sf}*=\mathrm{MAP}\left(F_{v,t},M_{s}\left(m==1\right)\right)⊕P_{sf} \end{array}$$

(8) $$\begin{array}{c} P_{sb}*=\mathrm{MAP}\left(F_{v,t},M_{s}\left(m==0\right)\right)⊕P_{sb} \end{array}$$

The support prototype is generated using the features of the support image. Even if the support and query images belong to the same class, the support prototype may ignore local information and result in prototype bias, which can adversely affect the performance of the query image. To tackle the problem of prototype bias, we generate an adaptive query prototype by combining the foreground–background prototype features, the query image features, and the multi-modal support prototype. We then use this query prototype to guide the segmentation of the query image. As seen in previous works, the ground truth mask of the image is required to generate the prototype. However, the ground truth mask of the query image is not available during the inference process. Therefore, we use the estimated mask of the query image instead of the ground truth mask to generate the query prototype. According to Equation (9), we compute the similarity of the previously obtained foreground prototype $P_{sf}*$ and background prototype $P_{sb}*$ with the query image feature $F_{q}$, respectively. The obtained results are passed through the ***softmax*** activation function to obtain the predicted mask ${\hat{M}}_{q}$.

(9) $$\begin{array}{c} {\hat{M}}_{q}=\mathrm{softmax}\left(\mathrm{cosine}\left(P_{\mathrm{sf}}*,P_{\mathrm{sb}}*,\;F_{q}\right)\right) \end{array}$$

To ensure that the adaptive query prototype accurately reflects the class characteristics of the query image, we define a specific threshold value of β to separate the estimated query image mask into foreground and background regions. This helps to ensure that the adaptive query prototype captures the class characteristics of the query image. We classify the predicted pixels as foreground when their values are >$\beta_{fg}$ and as background when they are >$\beta_{bg}$. According to the ablation experiments in Section 4, we conclude that for the foreground prototype $\beta_{fg}=0.7$, and for the background prototype $\beta_{bg}=0.6$. Thus, there is {$M_{qf}$, $M_{qb}$}, which will be used to generate the adaptive query prototype. As the foreground of the image is the primary object for segmentation by the model, the information contained in the foreground pixels is relatively distinct. We utilize the MAP function to merge the query image features with the predicted foreground pixels, creating an adaptive foreground query prototype (as in Equation (10)).

(10) $$\begin{array}{c} AP_{qf}=\mathrm{MAP}\left(F_{q},{\hat{M}}_{qf}\right) \end{array}$$

**Figure 4:**
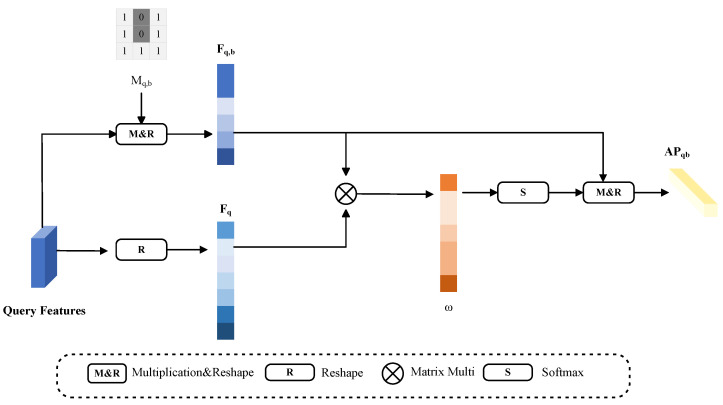
The generation process of adaptive background prototype. We exploit the predicted query mask to obtain a similarity matrix. Then, we use the matrix and query features to generate an adaptive background prototype.

The background of the image contains more complex information than the foreground, which can significantly impede the FSS task. Previous methods generate multiple prototypes using background pixels and then select the prototype with the highest similarity to match with the query image features. However, this approach is not only time consuming but also inaccurate. We propose generating an adaptive background query prototype in this paper. Based on Figure 4, we merge the estimated background query mask $M_{qb}$ with the query feature $F_{q}$ through matrix multiplication. We then modify the shape of the feature map using the reshape operation to acquire $F_{qb}$. To activate each background pixel in the feature map, we create a matrix ω by performing multiplication between $F_{qb}$ and the query feature $F_{q}$ (as in Equation (11)). Finally, we multiply ω by the softmax activation function with $F_{qb}$ to acquire the adaptive background query prototype ${\mathrm{AP}}_{\mathrm{qb}}$ (as in Equation (12)). Attaching the query image features to each background pixel helps prevent imprecise segmentation results caused by the cluttered information in the background pixels. This approach aids in creating an adaptive background query prototype that more accurately reflects the class characteristics of the query image.

(11) $$\begin{array}{c} \omega =\mathrm{Matmul}\left(F_{qb},F_{q}\right) \end{array}$$

(12) $$\begin{array}{c} {\mathrm{AP}}_{\mathrm{qb}}=\mathrm{Matmul}\left(F_{\mathrm{qb}},\mathrm{softmax}\left(\omega \right)\right) \end{array}$$

Now that we have the adaptive query prototype $AP_{q}$ {$AP_{qf}$, $AP_{qb}$}, Section 3.5 will describe the specific approach for designing the loss function to effectively utilize the adaptive query prototype.

### 3.5. Multi-Prototype Matching Loss Function

Through the introduction of the previous sections, we arrived at the multi-modal support prototype $P_{s}^{*}\left\{P_{sf}^{*},P_{sb}^{*}\right\}$ and the adaptive query prototype $AP_{q}\left\{AP_{qf},AP_{qb}\right\}$. We used these two prototypes to add up and balance the proportion of the two types of prototypes by coefficients. The final generated prototype is shown in Equation (13).

(13) $$\begin{array}{c} P_{M}=\alpha_{1}P_{s}^{*}+\alpha_{2}AP_{q} \end{array}$$

In our experiments, we set the two coefficients $\alpha_{1}=\alpha_{2}=0.5$ and use the final generated prototype $P_{M}$ to compute the similarity with the query feature $F_{q}$ to derive the final prediction mask. According to Equation (3), the final prototype $P_{M}$ and query image features are used to obtain the final prediction mask ${\hat{M}}_{2}$ by the cosine similarity function and softmax function. We use a binary cross-entropy (BCE) loss function to evaluate the gap between the prediction mask and ground truth mask of the image to continuously update the model parameters (as in Equation (14)).

(14) $$\begin{array}{c} L_{1}=\mathrm{BCE}\left(M_{2},M_{q}\right)) \end{array}$$

To be able to take full advantage of the adaptive query prototype and the multi-modal support prototype, we calculate the similarity between these two prototypes and the query image separately and derive the prediction mask. The loss function is then used to calculate the value of the prediction mask with respect to ground truth mask (as in Equations (15) and (16)).

(15) $$\begin{array}{c} L_{2}=\mathrm{BCE}\left(\mathrm{cosine}\left({\mathrm{AP}}_{q},F_{q}\right),M_{q}\right) \end{array}$$

(16) $$\begin{array}{c} L_{3}=\mathrm{BCE}\left(\mathrm{cosine}\left(P_{s}^{*},F_{s}\right),M_{s}\right) \end{array}$$

Finally, we use the weight coefficients λ to balance the weight of the three loss functions (as in Equation (17)). In our experiments we set $\lambda_{1}=\lambda_{2}=1$ and $\lambda_{3}=0.2$.

(17) $$\begin{array}{c} L=\lambda_{1}L1+\lambda_{2}L2+\lambda_{3}L3 \end{array}$$

## 4. Experiments

### 4.1. Datasets and Implementation Details

**Datasets.** We conduct experiments on two benchmark datasets, namely PASCAL-$5^{i}$ [62] and COCO-$20^{i}$ [2], where the PASCAL dataset was used as a benchmark for evaluating the performance of different image segmentation methods. The dataset contains images of 20 different object classes, each labeled at the pixel level, meaning that each pixel is labeled according to the object to which it belongs. We follow previous work and divide the 20 categories in the PASCAL dataset into four folds, each containing five categories. We use three folds for training and one fold for inference, ensuring that the training set and the test set do not intersect in FSS task. To ensure the validity of the experiment, we use fold0 for inference when the remaining three folds are used for training, and use fold1 for inference when other fold is used for training. We repeat these experiments four times and report the performance of each fold separately. The COCO dataset contains over 330,000 images, featuring more than 80 different types of objects commonly found in complex real-world scenes. Compared to PASCAL, the COCO dataset is a significantly more challenging task with much greater category and image scene complexity. In such a challenging task, our method can still achieve good performance. We similarly followed the setup of previous work by dividing the 80 classes in the COCO into four folds and reporting the scores on each fold separately.
**Implementation Details.** We used the classical ResNet-50/101 [4] as the backbone network and utilized the pretraining parameters on ImageNet [1]. As CLIP is on the text side, we use VITB-32 as the backbone network. We cropped the original image and ground truth mask to size (473,473). During training, we used stochastic gradient descent with a momentum of 0.9 and an initial learning rate of 0.001 to optimize the model parameters. During training, we use meta-learning to train the model. As described in Section 3.1, our model is trained with 24,000 episodes, each containing one support–query pair. We set one round of training with 1200 episodes, totaling 20 rounds of training per batch of data, with each batch containing four support–query pairs. We randomly selected 1000/4000 support–query pairs for testing, and the ground truth masks of the images were not visible during testing. Consistent with most previous work, we used mean Intersection-over-Union (mIoU) to report the model’s performance on both datasets. The formula for mIoU is shown in Equation (18),
  (18) $$mIoU=\frac{1}{n}\sum_{i=1}^{n}\frac{TP_{i}}{TP_{i}+FP_{i}+FN_{i}}$$
  where *n* is the number of classes, *i* denotes class *i*, *TP_i_* denotes the number of pixels correctly predicted as class *i*, *FP_i_* denotes the number of pixels that the model incorrectly predicts as class *i* for pixels that are not class *i*, and *FN_i_* denotes the number of pixels that the model incorrectly predicts as non-class *i* pixels.

### 4.2. Comparison with Previous Works

**PASCAL-$5^{i}$.** To verify the effectiveness of our proposed method, we compared our model with different approaches on the PASCAL and COCO datasets. As shown in Table 1, our model outperforms previous approaches significantly in both the one-shot and five-shot settings. In the one-shot experimental setting, the feature encoder using ResNet-50 exceeds the results of SSP [51] by 2.0% on average across the four folds. This demonstrates the effectiveness of the MSP and AFBM modules. While our current results in the one-shot experimental setting show a 1.1% decrease compared to HSNet, we have observed higher performance in the five-shot setting when compared to HSNet. We contend that this discrepancy arises due to the fact that HSNet utilizes an encoder–decoder architecture, which requires a longer training time compared to our proposed method. As stated in Table 2, the training time for HSNet is reported to be 54 h, whereas our proposed method requires only 5 h of training in the same experimental setting. Under the five-shot experimental setting, using ResNet-50 as the backbone network, we improved the scores of fold1 and fold2 to 73.0% and 75.1%, respectively, which is significantly ahead of previous work. After using the stronger ResNet-101 backbone network, we achieved even higher scores, with a score of 67.8% in fold0 and an average score of 65.9% across all four folds in the one-shot setting. In the five-shot setting, we improved the score of fold0 to 72.8% and the average score across all four folds to 74.5%, which is 4.1% higher than HSNet [55]. The few-shot segmentation model based on the prototype structure uses non-parametric measures, such as similarity functions, to calculate segmentation results, resulting in fast calculation and reasoning times. Although we use VITB-32 as the text feature encoder, this does not significantly increase training and inference times.

**Table 1:** Quantitative comparison results on PASCAL-$5^{i}$ dataset. The best and second best results are highlighted with **bold** and underline, respectively.

Method	Backbone			1-shot					5-shot		
		**fold0**	**fold1**	**fold2**	**fold3**	**Mean**	**fold0**	**fold1**	**fold2**	**fold3**	**Mean**
PANet [8]		44.0	57.5	50.8	44.0	49.1	55.3	67.2	61.3	53.2	59.3
PPNet [9]		48.6	60.6	55.7	46.5	52.8	58.9	68.3	66.8	58.0	63.0
PFENet [53]		61.7	69.5	55.4	56.3	60.8	63.1	70.7	55.8	57.9	61.9
CWT [63]	Res-50	56.3	62.0	59.9	47.2	56.4	61.3	68.5	68.5	56.6	63.7
HSNet [55]		**64.3**	70.7	60.3	**60.5**	**64.0**	**70.3**	**73.2**	67.4	**67.1**	69.5
MLC [64]		59.2	**71.2**	65.6	52.5	62.1	63.5	71.6	71.2	58.1	66.1
SSP [51]		61.4	67.2	65.4	49.7	60.9	68.0	72.0	74.8	60.2	68.8
**Ours**		63.5	67.8	**67.9**	52.2	62.9	69.2	73.0	**75.1**	61.4	**69.7**
FWB [7]		51.3	64.5	56.7	52.2	56.2	54.8	67.4	62.2	55.3	59.9
PPNet [9]		52.7	62.8	57.4	47.7	55.2	60.3	70.0	69.4	60.7	65.1
PFENet [53]		60.5	69.4	54.4	55.9	60.1	62.8	70.4	54.9	57.6	61.4
CWT [63]	Res-101	56.9	65.2	61.2	48.8	58.0	62.6	70.2	68.8	57.2	64.7
HSNet [55]		67.3	**72.3**	62.0	63.1	**66.2**	71.8	74.4	67.0	**68.3**	70.4
MLC [64]		60.8	71.3	61.5	56.9	62.6	65.8	74.9	71.4	63.1	68.8
SSP [51]		63.7	70.1	66.7	55.4	64.0	70.3	76.3	77.8	65.5	72.5
**Ours**		**67.8**	71.2	**67.7**	**57.1**	65.9	**72.8**	**76.7**	**81.7**	66.7	**74.5**
**COCO-$20^{i}$.** This is a very challenging dataset that contains 80 categories and more complex foreground–background relationships, but our proposed method still achieves better results than previous work. As in Table 3, under the one-shot setting with ResNet-50, our model achieved an average score 1.4% higher than SSP [51] and 1.1% higher than MLC [64] across all four folds. In the five-shot setting, we achieved a score of 56.5% in fold0, which is better than most previous approaches. When we used the stronger ResNet-101 backbone network, our model performed even better on complex datasets. In the one-shot setting, our model outperformed SSP [51] by 1.9% on average across all four folds, while in the five-shot setting, we outperformed it by 3.2% on average.

### 4.3. Efficiency Comparison with Previous Works

In our comparison with recent few-shot segmentation methods, we have observed that while our experimental results may be slightly lower than state-of-the-art methods, our method offers a significant advantage in terms of training time. Table 2 presents an efficiency comparison with previous state-of-the-art methods. As can be seen, compared to previous methods [55,66,67], although the method of HDMNet [67] significantly improves the performance, it takes much longer training time. As evident from Table 2, our proposed method demonstrates significantly lower training time compared to the method listed in the table. The fact that our proposed method requires only 5 h of training time serves as strong evidence of its effectiveness. The substantial reduction in training time significantly highlights the efficiency and capability of our method. It demonstrates our method’s ability to achieve results within a relatively short period of time.

### 4.4. Ablation Studies

**Figure 5:**
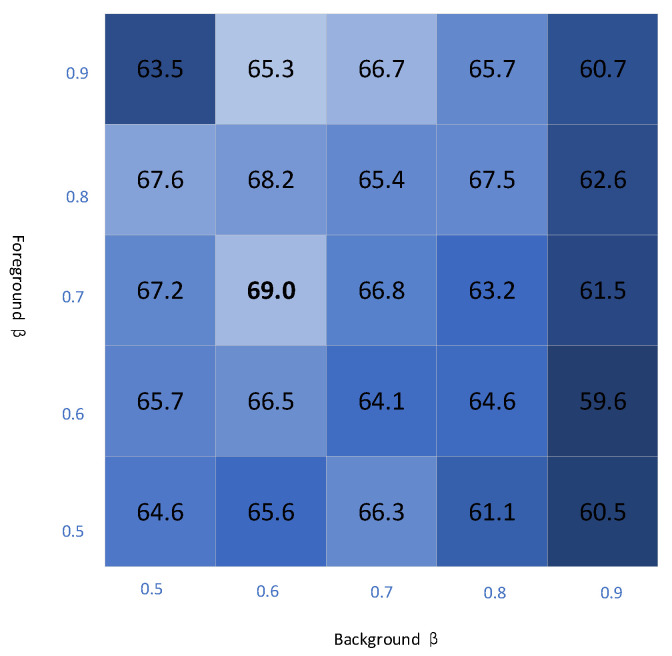
Ablation results for β, shades of color represent different performance.

**Ablation experiments for different modules.** To assess the effectiveness of our methods, we conducted ablation studies on the proposed MSP, AFBM and MML methods. These ablation experiments were conducted using a five-shot setting, and we utilized the ResNet-50 as the backbone network. As shown in Table 4, the proposed MSP improved the model’s performance by 1.6% compared to the baseline. This provides evidence that our proposed multi-modal support prototype effectively improve the model’s predictive capability, and the introduced textual features enhance the support prototype’s ability to recognize a novel class. AFBM further enhanced the model’s performance, improving the average performance by 3% compared to the baseline. We found that combining MSP with AFBM resulted in a significant performance improvement of the model, with the performance increasing to 68.7%, which is 5.6% higher than the baseline. Finally, by incorporating all methods, including the MML loss function, the model’s score increased significantly to 69.7% compared to the baseline of 6.6%. This result demonstrates the effectiveness of our proposed method.
**Ablation experiments for β.** We used $\beta_{fg}$ and $\beta_{bg}$ to generate the predicted mask ${\hat{M}}_{q}$ (as in Equation (9)). The choice of foreground and background thresholds in an image can significantly impact its performance. The threshold size determines which pixels are categorized as foreground or background, which, in turn, affects the accuracy and level of detail in the resulting segmentation. If the threshold is set too high, it will likely result in some foreground pixels being incorrectly assigned to background categories. Conversely, if the threshold is set too low, it will likely result in some background pixels being incorrectly assigned to the foreground category. We conduct ablation experiments for each value of $\beta_{fg}\in \left[0.5,0.9\right]$, $\beta_{bg}\in \left[0.5,0.9\right]$, and the results of the experiments are shown in Figure 5. Lighter colors represent better results, and darker colors represent worse results. Figure 5 summarizes the prediction scores of the model under different foreground and background thresholds, and the model predicts best when $\beta_{fg}=0.7$, $\beta_{bg}=0.6$.

### 4.5. Visualization Qualitative Results

**Figure 6:**
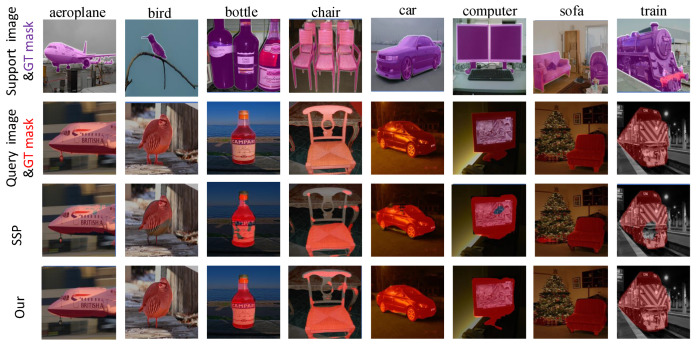
Example results on PASCAL-$5^{i}$ for different models. From top to bottom, we show the support image with ground truth mask region, query image with ground truth mask region, the SSP prediction and our prediction.

We present visualization qualitative results in a five-shot setting with the ResNet-101 backbone for better performance. As shown in Figure 6, the first line represents the support image and its ground truth mask. The second line represents the query image which the model aims to segment and its ground truth mask. The third row shows the segmentation result obtained by the SSP [51] method, while the last row shows the segmentation performance obtained by our proposed method. As can be seen, the predictions reveal that some of the target objects are not fully segmented (e.g., the body of the bird in the second column, and the head of the train in the last column). Additionally, there are instances where a part of the target object is incorrectly segmented (e.g., the Christmas tree in the seventh column is mistakenly identified as a sofa). In comparison to the SSP [51] approach, generating a multi-modal support prototype using text features is more accurate in recognizing different classes than the prototype generated using only image features.

## 5. Conclusions

In this paper, we propose to leverage CLIP to extract text features and utilize them as training samples to participate in the model’s training process. Text samples are incorporated into the training process through a very simple way that does not require additional training time. We also propose MSP to further leverage text features. MSP outperforms single-modal prototype features in accurately representing the semantic information of both images and text for a given object class. Additionally, we introduce the AFBM module, which utilizes the foreground and background information of an image to generate adaptive query prototypes for images. In order to align the proposed adaptive query prototype with the multi-modal support prototypes, we have developed new loss functions to maximize the utilization of different prototypes. Finally, we train the model by combining multiple prototypes via the MML loss function and achieve good scores on two generalized datasets, PASCAL-$5^{i}$ and COCO-$20^{i}$. Our proposed method exhibits a significantly reduced model training time compared to previous models, highlighting the effectiveness of our approach. This demonstrates a favorable balance between the training time and the results of our method. By combining CLIP with a prototype-structured FSS model, we further explore the potential of CLIP in FSS tasks. We hope that our work can provide valuable insights for future research endeavors aimed at addressing issues related to a multi-modal pretrained model.

## Figures and Tables

**Table 2:** Efficiency comparison with ResNet-50 on PASCAL-$5^{i}$ in 1-shot setting.

Method	mIoU	Training	Inference
PFENet [53]	60.8	24 h	52 ms
CWT [63]	56.3	10 h	232 ms
MMNet [65]	61.8	64 h	128 ms
HSNet [55]	64.0	54h	101 ms
BAM [66]	64.6	21 h	50 ms
HDMNet [67]	69.4	20 h	56 ms
Ours	62.9	5 h	60 ms

**Table 3:** Quantitative comparison results on COCO-$20^{i}$ dataset. The best and second best results are highlighted with **bold** and underline, respectively.

Method	Backbone			1-shot					5-shot		
		**fold0**	**fold1**	**fold2**	**fold3**	**Mean**	**fold0**	**fold1**	**fold2**	**fold3**	**Mean**
FWB [7]		16.9	17.9	20.9	28.8	21.1	19.1	21.4	23.9	30.0	23.6
PANet [8]		31.5	22.6	21.5	16.2	23.0	45.9	29.2	30.6	29.6	33.8
PPNet [9]		36.5	26.5	26.0	19.7	27.2	48.9	31.4	36.0	30.6	36.7
CWT [63]	Res-50	32.2	36.0	**31.6**	**31.6**	32.9	40.1	43.8	**39.0**	**42.4**	41.3
MLC [64]		46.8	35.3	26.2	27.1	33.9	54.1	41.2	34.1	33.1	40.6
SSP [51]		46.4	35.2	27.3	25.4	33.6	53.8	41.5	36.0	33.7	41.3
**Ours**		**48.3**	**36.5**	28.9	26.5	**35.0**	**56.5**	**43.9**	38.0	35.6	**43.5**
PFENet [53]		34.3	33.0	32.3	30.1	32.4	38.5	38.6	38.2	34.3	37.4
PMMs [68]		29.5	36.8	28.9	27.0	30.6	33.8	42.0	33.0	33.3	35.5
SCL [69]		36.4	38.6	**37.5**	**35.4**	37.0	38.9	40.5	41.5	38.7	39.9
CWT [63]	Res-101	30.3	36.6	30.5	32.2	32.4	38.5	46.7	39.4	43.2	42.0
MLC [64]		50.2	37.8	27.1	30.4	36.4	57.0	46.2	37.3	37.2	44.4
SSP [51]		50.4	39.9	30.6	30.0	37.7	57.8	47.0	40.2	39.9	46.2
**Ours**		**52.3**	**40.7**	33.7	31.7	**39.6**	**61.5**	**48.4**	**42.7**	**43.4**	**49.0**

**Table 4:** Ablation studies for different modules.

MSP	AFBM	MML	fold0	fold1	fold2	fold3	Mean
			60.2	69.1	70.0	53.0	63.1
✓			62.5	70.2	71.8	54.3	64.7 _↑1.6_
	✓		65.7	71.3	72.0	56.5	66.4 _↑3.3_
✓	✓		68.4	72.4	73.6	60.2	68.7 _↑5.6_
✓	✓	✓	69.2	73.0	75.1	61.4	69.7 _↑6.6_

## Data Availability

The data are contained within the article.
